# Nitrogen uptake and nitrogen fertilizer recovery in old and modern wheat genotypes grown in the presence or absence of interspecific competition

**DOI:** 10.3389/fpls.2015.00185

**Published:** 2015-03-25

**Authors:** Paolo Ruisi, Benedetto Frangipane, Gaetano Amato, Alfonso S. Frenda, Antonella Plaia, Dario Giambalvo, Sergio Saia

**Affiliations:** ^1^Dipartimento di Scienze Agrarie e Forestali, Università degli Studi di PalermoPalermo, Italy; ^2^Centro di Sperimentazione e Certificazione delle SementiBattipaglia, Italy; ^3^Dipartimento di Scienze Economiche, Aziendali e Statistiche, Università degli Studi di PalermoPalermo, Italy

**Keywords:** durum wheat, old genotypes, modern varieties, nitrogen assimilation capacity, weed suppression ability, weed competition, 15N fertilizer recovery

## Abstract

Choosing genotypes with a high capacity for taking up nitrogen (N) from the soil and the ability to efficiently compete with weeds for this nutrient is essential to increasing the sustainability of cropping systems that are less dependent on auxiliary inputs. This research aimed to verify whether differences exist in N uptake and N fertilizer recovery capacity among wheat genotypes and, if so, whether these differences are related to a different competitive ability against weeds of wheat genotypes. To this end, 12 genotypes, varying widely in morphological traits and year of release, were grown in the presence or absence of interspecific competition (using *Avena sativa* L. as a surrogate weed). Isotopic tracer ^15^N was used to measure the fertilizer N uptake efficiencies of the wheat genotypes and weed. A field experiment, a split-plot design with four replications, was conducted during two consecutive growing seasons in a typical Mediterranean environment. In the absence of interspecific competition, few differences in either total N uptake (range: 98–112 kg N ha^−1^) or the ^15^N fertilizer recovery fraction (range: 30.0–36.7%) were observed among the wheat genotypes. The presence of competition, compared to competitor-free conditions, resulted in reductions in grain yield (49%), total N uptake (29%), and an ^15^N fertilizer recovery fraction (32%) that were on average markedly higher in modern varieties than in old ones. Both biomass and grain reductions were strongly related to the biomass of the competitor (correlation coefficients > 0.95), which ranged from 135 to 573 g m^−2^. Variations in both grain and biomass yield due to interspecific competition were significantly correlated with percentage of soil cover and leaf area at tillering, plant height at heading, and total N uptake, thus highlighting that the ability to take up N from the soil played a certain role in determining the different competitive abilities against weed of the genotypes.

## Introduction

Breeding activity on durum wheat (*Triticum durum* Desf.) carried out in Italy over the past 70 years has involved the constant release of cultivars selected to perform well under intensive crop management and characterized by an increased yield potential (De Vita et al., 2010), also through the exploitation of *Rht* genes, which has caused a drastic reduction in plant height and a consequent increase in the harvest index (Giunta et al., 2007). This has resulted in a reduction in the competitive ability of wheat against weeds (Lemerle et al., 1996; Vandeleur and Gill, 2004). Such a trait is particularly desired in varieties used in organic and low-input farming systems (Löschenberger et al., 2008). Differences in cultivars in terms of either weed suppression ability (i.e., the ability of a cultivar to reduce weed growth through competition) or weed tolerance (i.e., the ability of a cultivar to achieve high yields despite weed competition) have been observed in wheat: older cultivars are generally more competitive against weeds than higher yielding, semi-dwarf modern cultivars (Korres and Froud-Williams, 2002; Mason et al., 2007; Hoad et al., 2008). The magnitude of the differences in either weed suppression ability or weed tolerance can be impressive. The phenotypic trait most commonly associated with competitive ability against weeds is plant height (Lemerle et al., 2001; Murphy et al., 2008; Zerner et al., 2008), as growing taller than the neighbors results in more available light and the ability to shade out competitors. Other traits, such as vigorous growth, rapid ground covering, canopy structure, and overall leaf area, may also influence crop competitive ability (as recently reviewed by Worthington and Reberg-Horton, 2013). Moreover, some studies suggest that allelopathy may play an important role in weed suppression in wheat (Bertholdsson, 2005; Fragasso et al., 2013). However, competitive ability depends not on a single trait alone but on the interaction of several traits (Eisele and Köpke, 1997; Mason and Spaner, 2006). It is possible that an important role could be played by the capacity of the crop to compete with weeds for belowground resources, such as water and nutrients, and in particular for nitrogen (N).

As concerns wheat, many researchers have shown that great differences exist among genotypes in N use efficiency, but it is still unclear whether and in which way breeding activity influences this trait. Austin et al. (1977) found a general reduction in total wheat N uptake for semi-dwarf lines compared with taller ones. Other authors (Ortiz-Monasterio et al., 1997; Le Gouis et al., 2000; Brancourt-Hulmel et al., 2003; Guarda et al., 2004; Sylvester-Bradley and Kindred, 2009) found that N uptake was significantly greater for new varieties compared to older ones, indicating that N capture has increased through breeding, regardless of the amount of N fertilizer applied. In contrast, Foulkes et al. (1998) found that newer varieties were less efficient at acquiring soil N when no N fertilizer was applied, but they were more efficient than older ones in recovering fertilizer N when this nutrient was applied in an amount adequate to their potential (i.e., when the N fertilizer was applied at the optimum doses). However, other authors have found no relationship between N uptake and year of release of the variety (Calderini et al., 1995; Motzo et al., 2004; Giambalvo et al., 2010). Possible explanations for these discrepancies include the different genotypes used, the different crop management schemes applied (e.g., the timing and method of application of N fertilizer), and the different climatic and soil conditions (i.e., water availability, soil fertility, and N availability) in which these studies were performed. To date, the identification of genotypes highly efficient in N use is one of the foremost objectives of organic wheat breeding programs (Hoad et al., 2012).

To the best of our knowledge, no information is available on (i) whether wheat genotypes vary in their ability to compete with weeds for N or (ii) what effect different N uptake capacities can have on a genotype's ability to compete with weeds. Thus, the present study had as objectives to verify whether differences in N uptake and N fertilizer recovery exist among wheat genotypes that differ in their year of release and, if so, to verify whether these differences are related to a different competitive ability against weeds. To this end, 12 genotypes, chosen to include large variability in plant growth habit, grain yield potential, and year of release, were grown in the presence or absence of interspecific competition. Isotopic tracer ^15^N was used to measure the fertilizer N uptake efficiencies of the wheat genotypes and weed. Ultimately, the study aimed to obtain information useful for choosing or developing wheat varieties suitable for low-input systems (i.e., those less reliant on the use of both herbicides and fertilizers) or organic systems (i.e., varieties able to efficiently compete with weeds for N).

## Material and methods

### Experimental site

A field experiment was conducted during two consecutive growing seasons (2008–09 and 2009–10) at the experimental farm Pietranera, located about 30 km north of Agrigento, Italy (37°30′ N, 13°31′ E; 178 m asl). In both growing seasons, the soil was a Chromic Haploxerert with a clay texture (525 g kg^−1^ clay, 227 g kg^−1^ silt, and 248 g kg^−1^ sand; pH 8.2; 16.8 g kg^−1^ total C and 1.78 g kg^−1^ total N).

The climate of the experimental site is semiarid Mediterranean with a mean annual rainfall of 581 mm, concentrated mostly during the autumn–winter period (September–February; 76%), followed by spring (March–May; 19%). There is a dry period from May to September. The mean air temperatures are 15.9°C in autumn, 9.7°C in winter, and 16.5°C in spring. Weather data were collected from a weather station located within 200 m of the experimental site.

### Experimental design and crop management

The experiments were set up in a split-plot design with four replications. Main plots were planted with 12 wheat genotypes (11 of durum wheat, *T. durum* Desf.; 1 of bread wheat, *T. aestivum* L., Maiorcone) that varied widely in their morpho-phenological traits (Table 1 and Table S1). Out of these 12 genotypes, 5 were Sicilian wheat landraces that were collected from Sicilian farmers. An interspecific competition treatment (present [i.e., weedy] or absent [i.e., weed-free]) was set up in the subplots. The size of each subplot was 1.5 × 8.0 m (8 rows, each 8.0 m long, spaced at 0.18 m). Oat (*Avena sativa* L.) was chosen as a surrogate weed to obtain a homogenous weed density across the experimental plots. We used *A. sativa* as a surrogate weed rather than a real wild weed (e.g., *A. sterilis*, which is spontaneous and widespread in the experimental area) to ensure reliability and uniformity of emergence and synchronous development (according to Cousens et al., 2003). A variety with a medium-tall stature and with medium-late heading and maturity (Rogar 8) was used. In both growing seasons, the previous crop was berseem clover (*Trifolium alexandrinum* L.). Before the experiment began, the soil was plowed in August and harrowed after the first autumn rainfalls. Phosphate fertilizer was applied before sowing at 69 kg P_2_O_5_ ha^−1^ as triple superphosphate. In both years, plots were sown at the end of December, using 350 germinable wheat seeds per square meter. In the relevant subplots (i.e., weedy plots), oat was seeded in the same row as the wheat to maximize interspecific competition. Oat was planted at 100 germinable seeds per square meter. Ammonium sulfate fertilizer was applied at the time of seed emergence. Plots were labeled with ^15^N fertilizer (80 kg N ha^−1^ as (NH_4_)_2_SO_4_ with an isotopic enrichment of 1.33 atom%) added to a 1.20-m^2^ (6 rows, 1.10 m long, 0.18 m apart) area in the middle of each subplot, following the application procedure described by Høgh-Jensen and Schjoerring (1994); the rest of the subplots (outside of the ^15^N-labeled area) received equivalent amounts of unlabeled fertilizer. All natural weeds in both weedy and weed-free plots were removed by hand.

**Table 1:** **Wheat genotypes tested by year of release, pedigree, and some agronomic traits**.

**Genotypes**		**Year of release**	**Group**	**Plant stature**	**Heading time**	**Pedigree**
1	Biancuccia	—	Old	Tall	Late	Indigenous landrace from Sicily
2	Maiorcone	—	Old	Tall	Late	Indigenous landrace from Sicily
3	Realforte	—	Old	Tall	Late	Indigenous landrace from Sicily
4	Russello	—	Old	Tall	Late	Indigenous landrace from Sicily
5	Scorsonera	—	Old	Tall	Late	Indigenous landrace from Sicily
6	Cappelli	1915	Old	Tall	Late	Selection from North-African landrace
7	Capeiti 8	1955	Modern	Mid	Early	Eiti 6/Cappelli
8	Creso	1974	Modern	Short	Late	Yaktana-54/Norin 10-B//2^*^Cappelli-63/3/3^*^Tehuacan-60/4/Cappelli-B144
9	Simeto	1988	Modern	Short	Early	Capeiti 8/Valnova
10	Valbelice	1992	Modern	Mid	Early	0111/BC-5
11	Iride	1996	Modern	Short	Early	Altar-84/Ares
12	Claudio	1998	Modern	Short-mid	Early-mid	CIMMYT's selection 35/Durango//IS1938/Grazia

### Measurements

At the end of tillering and at heading, a 1-m^2^ portion inside each subplot was sampled, and plants were sorted by species (wheat and oat). Plants and tillers of each species were counted and separated into leaves, stems, and spikes; the fresh weight of each sample was determined and the leaf area of the leaves was immediately measured using a leaf area meter (LI-COR LI-3100C Area Meter). All samples were dried in a forced air oven at 60°C for 36 h and weighed. The plant height, percentage of soil cover (visual estimation by two independent observers), and lodging (as a percentage of the part of the plot that was lodged; visual estimation by two independent observers) for each wheat genotype grown in both the presence and absence of interspecific competition were also recorded. Moreover, leaf habit (visual score: 1, erect; 5, horizontally disposed; Kruepl et al., 2006) was recorded in weed-free conditions only.

At seed maturity, the area of each subplot labeled with ^15^N was sampled, and plants were sorted by species, oven dried at 60°C for 36 h, weighed, ground to a fine powder (sieved using a 0.1-mm mesh size) in a fast running mill (Retsch ZM 100), and analyzed for total N and ^15^N enrichment (using an elemental analyzer-isotope ratio mass spectrometer, Carlo Erba NA1500). Plant height, lodging, grain yield and yield components (number of spikes per square meter, number of seeds per spike, and 1000-seed weight), and N grain content were recorded for both wheat and oat.

### Calculation and data analysis

For both wheat and oat, data on ^15^N enrichment of biomass were used to calculate the labeled-fertilizer N recovery (^15^N_REC_) on an area basis (kg N ha^−1^) and a percentage basis, according to Hauck and Bremner (1976):

$$\begin{array}{l} {}^{15}N_{REC}=Nt\times \frac{{}^{15}Nfp{-}^{15}Nnfp}{{}^{15}Nfert{-}^{15}Nnfp\;}\text{and}\\ {\%}^{15}N_{REC}=\;\frac{{}^{15}N_{REC}}{f}\times 100 \end{array}$$

where *Nt* was the plant N content measured at maturity (kg ha^−1^), *^15^Nfp* was the atom% ^15^N in the fertilized plants, *^15^Nnfp* was the atom% ^15^N in the unfertilized plants, *^15^Nfert* was the atom% ^15^N in the fertilizer, and *f* was the fertilizer rate (kg N ha^−1^).

For all measured variables normality was tested using Shapiro–Wilk test of normality. All variables corresponding to proportions were arcsine transformed before analysis to ensure a better fit with the Gaussian law distribution. Data from each year were analyzed separately, and homogeneity of variances was assessed using Bartlett's test before combined analyses were performed.

Two separate analyses were performed for each measured variable. In the first one, data were analyzed according to a split-plot design with genotype (G) as the main-plot treatment, interspecific competition (IC) as a subplot factor, and year and replicates as random factors. Considering also that G can be aggregated into two groups [Old (released before 1915) vs. Modern; Table 1], we performed a second analysis with Group and IC as fixed factors and, again, year and replicates as random factors. To compare genotype means, we computed Fisher's least significant difference (LSD; 5% probability level) differently in the presence or absence of interaction between G and IC. In the absence of interaction, marginal G means can be compared (i.e., averaged over IC levels), and the appropriate LSD is

$$LSD=t_{0.05,v}\sqrt{\frac{2E_{G}}{rtb}}\text{ with }v=\left(rt-1\right)\left(g-1\right).$$

In the presence of interaction between G and IC, G means at the same or different IC levels can be compared, and the appropriate LSD is

$$\begin{array}{l} LSD=t_{0.05,v}\sqrt{\frac{2[(b-1)E_{GxIC}+E_{G}]}{rtb}}\text{ with}\\ t_{0.05,v}=\frac{(b-1)E_{GxIC}t_{0.05,v1}+E_{G}t_{0.05,v2}}{(b-1)E_{GxIC}+E_{G}},\\ \;v_{1}=g(rt-1)(b-1),v_{2}=(rt-1)(g-1). \end{array}$$

Finally a Pearson correlation analysis was performed to highlight the relationships among morpho-agronomic traits (measured at tillering, heading, and maturity in the weed-free plots) and competitive ability against weeds (measured either as percent decreases in grain and biomass yields in weedy compared to weed-free plots or as weed biomass in weedy plots). All analyses were carried out in the R environment (R Development Core Team, 2011).

## Results

### Weather conditions

**Figure 1:**
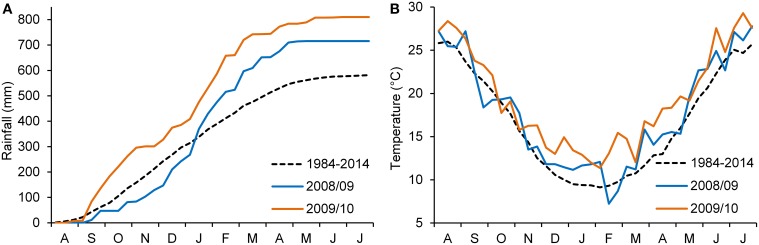
**Accumulated rainfall**. **(A)** 10-day mean air temperature **(B)** at the experimental site during the two growing seasons (2008–2009 and 2009–2010); 30-year average 10-day temperatures and accumulated rainfall are also included.

The weather conditions during the experimental period are shown in Figure 1. Total rainfall in 2008–09 was 715 mm, 23% higher than the long-term average for the area. About 400 mm of rainfall was recorded during the winter. Although this rainfall resulted in excessive water in the soil, it had no apparent effect on plant density or root disease. The mean monthly temperature during the experimental period was similar to the normal mean temperature. In 2009–10, total rainfall was 810 mm (39% higher than the long-term average for the area), concentrated mostly during the autumn–winter period (September–February; 81%). The mean monthly temperature was higher than average, particularly during winter and spring.

### Measurements at the end of tillering

**Figure 2:**
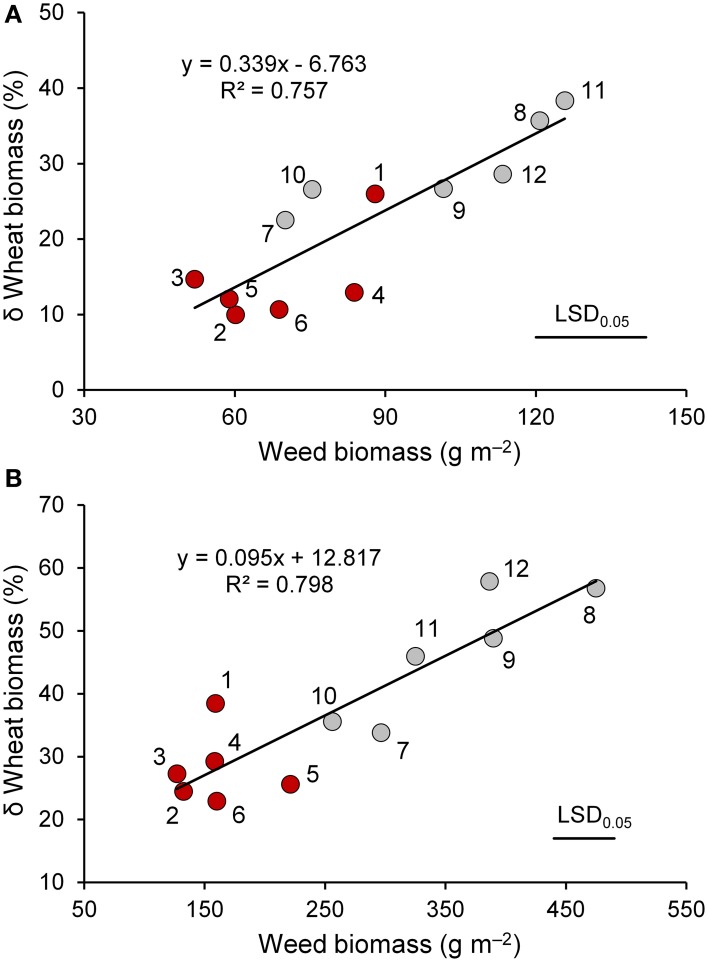
**The relationship between weed biomass and percent variation in biomass of wheat genotypes (red circle, “old” genotypes; gray circles, “modern” varieties) in the presence of interspecific competition with respect to the absence of competition at the end of tillering (A) and at heading (B)**. 1, Biancuccia; 2, Maiorcone; 3, Realforte; 4, Russello; 5, Scorsonera; 6, Cappelli; 7, Capeiti; 8, Creso; 9, Simeto; 10, Valbelice; 11, Iride; 12, Claudio.

Significant differences among wheat genotypes were observed in the percentage of soil cover, leaf habit, and leaf area index (LAI), with higher values observed in the old genotypes compared to the modern varieties (Table 2). However, no differences among genotypes were detected in the biomass production per unit area. Interspecific competition significantly affected all traits with the exception of leaf habit. On average, wheat biomass production per unit area decreased by 22% when a competitor was present, but varied responses were observed among genotypes. The magnitude of the decrease was related to the biomass production of the competitor, which was markedly higher in the modern varieties compared to the old genotypes (Figure 2A). The effects of interspecific competition on plant height varied by wheat genotype.

**Table 2:** **Wheat traits measured at the end of tillering in 12 genotypes grown in weed-free (WF) and weedy (W) conditions**.

**Genotype**	**Soil cover (%)**		**Plant height (cm)**		**Leaf habit^§^ (score: 1–5)**	**Plant biomass (g DM m^−2^)**		**LAI**	
	**WF**	**W**	**WF**	**W**	**WF**	**WF**	**W**	**WF**	**W**
**OLD**									
Biancuccia	77	78	43	42	3.0	207	153	2.01	1.31
Maiorcone	76	81	42	43	3.1	249	225	2.72	2.51
Realforte	83	81	45	43	3.8	267	228	2.92	2.21
Russello	76	81	40	42	4.4	209	182	1.98	1.70
Scorsonera	75	78	44	43	3.2	216	190	1.81	1.50
Cappelli	76	79	47	46	3.3	227	203	2.15	1.81
**MODERN**									
Capeiti 8	71	74	48	42	2.9	229	177	1.86	1.27
Creso	69	75	37	40	3.2	219	141	2.24	1.35
Simeto	68	77	41	39	2.8	222	163	1.83	1.19
Valbelice	78	79	52	46	3.5	242	178	1.88	1.35
Iride	69	76	40	39	2.8	202	125	1.69	1.01
Claudio	70	73	44	41	2.9	223	159	1.97	1.28
**SIGNIFICANCE LEVEL**									
Genotype (G)	^*^		^*^		^**^	ns		^**^	
Interspecific Compet. (IC)	^***^		^**^		—	^***^		^***^	
G × IC	ns		^**^		—	^*^		ns	
LSD_0.05_	6.2		6.0		0.27	59.2		0.678	
Old vs. Modern	^***^		ns		^**^	^*^		^***^	
(Old vs. Modern) × IC	ns		ns		—	^*^		ns	

*Data are the means of two growing seasons*.

§ *Leaf habit (visual score: 1, erect; 5, horizontally disposed) was recorded in weed-free conditions only*.

*, **, *** *significant at the 0.05, 0.01, and 0.001 probability level, respectively*.

*ns, not significant*.

### Measurements at heading

As expected, wheat genotypes differed markedly in heading date (Table 3). The modern varieties headed on average 2 weeks earlier than the old genotypes, with the exception of Creso, which had a heading date not different from that of the old genotypes. On average, old genotypes had a greater plant height and a higher biomass production compared to the modern varieties; the latter result appears to be related to their longer vegetative phase (defined as the interval between emergence and heading date). Interspecific competition greatly affected many traits. In particular, spike density, plant biomass, and LAI decreased on average by 24, 36, and 39%, respectively, when a competitor was present. For these three traits, the detrimental effect of interspecific competition varied significantly by wheat genotype (interaction genotype × interspecific competition was always significant at *P* < 0.05). For plant biomass, decreases due to the presence of a competitor were on average 27% for the old genotypes and 47% for the modern varieties. Differences observed among wheat genotypes for biomass production were related to the biomass production of the competitor, which ranged from 127 g m^−2^ (Realforte) to 475 g m^−2^ (Creso; Figure 2B).

**Table 3:** **Wheat traits measured at heading in 12 genotypes grown in weed-free (WF) and weedy (W) conditions**.

**Genotype**	**Heading time (days from 1 April)**		**Plant height (cm)**		**Lodging (%)**		**Spike density (n. m^−2^)**		**Plant biomass (g DM m^−2^)**		**LAI**	
	**WF**	**W**	**WF**	**W**	**WF**	**W**	**WF**	**W**	**WF**	**W**	**WF**	**W**
**OLD**												
Biancuccia	33	33	130	124	36	14	311	231	1087	669	2.14	1.43
Maiorcone	32	32	130	126	18	15	312	283	939	785	2.93	2.03
Realforte	31	31	129	119	19	19	350	314	974	708	2.95	2.16
Russello	33	33	151	142	8	8	253	214	963	681	2.36	1.35
Scorsonera	27	28	137	126	1	3	278	247	846	629	2.18	1.61
Cappelli	29	29	130	128	21	6	276	242	968	746	3.06	1.82
**MODERN**												
Capeiti 8	16	16	97	93	0	0	295	212	657	435	1.87	1.24
Creso	30	30	76	69	0	0	315	195	836	361	2.91	1.39
Simeto	19	19	71	69	0	0	259	150	691	354	2.23	1.33
Valbelice	15	15	104	100	0	0	306	240	723	466	2.32	1.50
Iride	16	17	70	64	0	0	327	210	623	337	1.99	1.16
Claudio	19	19	77	77	0	0	304	185	776	327	2.83	1.10
**SIGNIFICANCE LEVEL**												
Genotype (G)	^***^		^***^		^***^		^***^		^***^		^***^	
Interspecific Compet.(IC)	ns		^***^		^*^		^***^		^***^		^***^	
	ns		ns		^**^		^**^		^*^		^**^	
LSD_0.05_	1.1		9.2		13.6		39.1		133.9		0.519	
Old *vs*. Modern	^***^		^***^		^***^		^**^		^***^		^**^	
(Old *vs*. Modern) × IC	ns		ns		ns		^***^		^*^		ns	

*Data are the means of two growing seasons*.

*, **, *** *significant at the 0.05, 0.01, and 0.001 probability level, respectively*.

*ns, not significant*.

### Measurements at maturity

**Figure 3:**
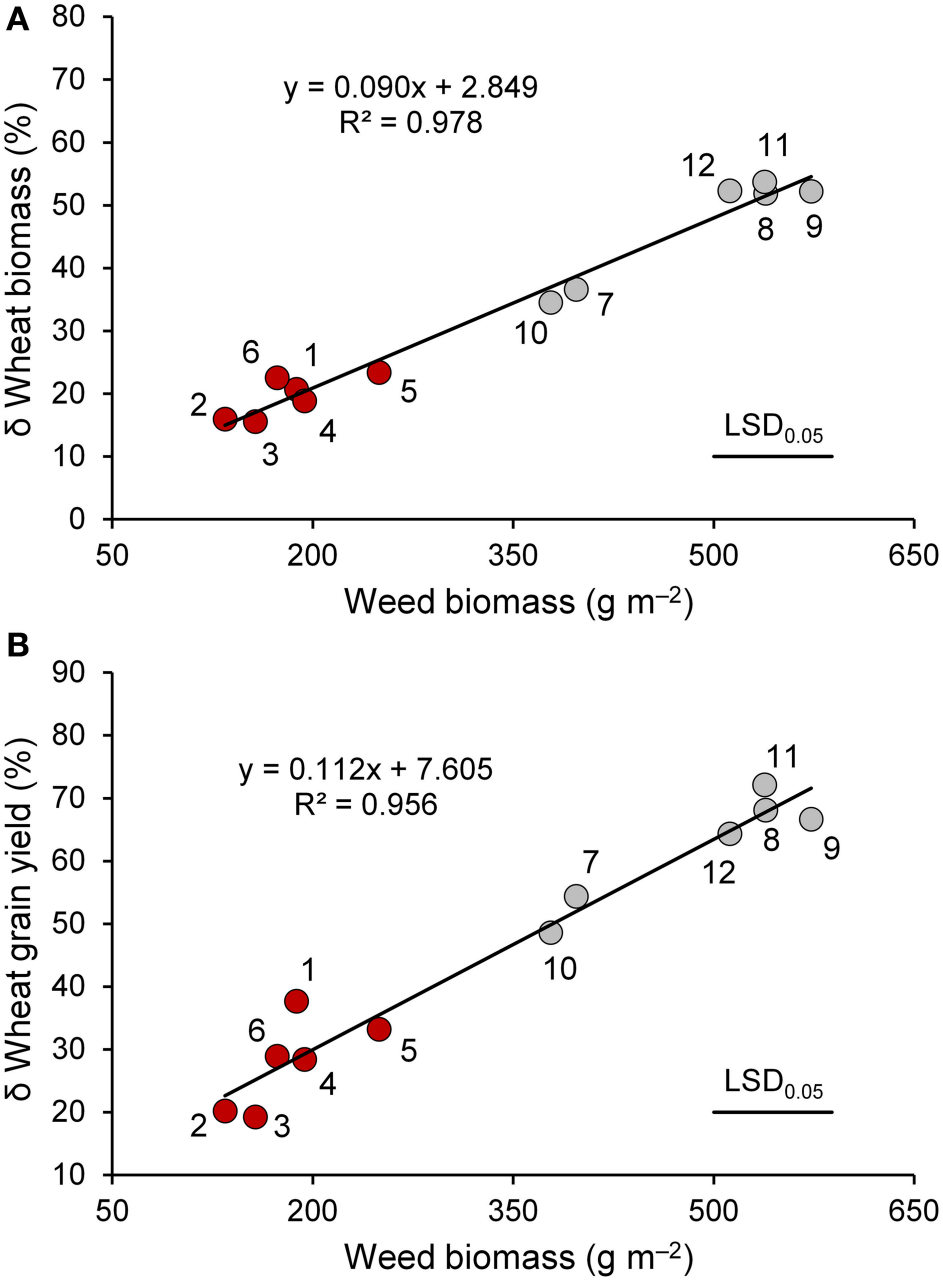
**The relationship between weed biomass and percent variation in biomass (A) and grain yield (B) of wheat genotypes (red circle, “old” genotypes; gray circles, “modern” varieties) in the presence of interspecific competition with respect to the absence of competition at maturity**. 1, Biancuccia; 2, Maiorcone; 3, Realforte; 4, Russello; 5, Scorsonera; 6, Cappelli; 7, Capeiti; 8, Creso; 9, Simeto; 10, Valbelice; 11, Iride; 12, Claudio.

Wheat genotypes differed widely in plant height (Table 4), which ranged on average from 76 cm (Simeto) to 152 cm (Russello). All genotypes taller than 100 cm were subjected to lodging that in some genotypes appeared to be particularly severe. Interspecific competition markedly reduced biomass and grain yields, harvest index, and 1000-seed weight; such decreases were greater in the modern varieties than in the old genotypes. In particular, interspecific competition caused strong reductions in the modern varieties (47 and 62%, respectively, for biomass and grain yield) and moderate reductions in the old genotypes (18 and 26%, respectively, for biomass and grain yield). As observed at both tillering and heading, both biomass, and grain reductions were related to the biomass of the competitor (Figure 3), which ranged from 135 g m^−2^ (Maiorcone) to 573 g m^−2^ (Simeto). On the whole, the surrogate weed accumulated more biomass when grown with the modern genotypes than the old ones. Among the modern varieties, the surrogate weed produced less biomass when grown with Valbelice (378 g m^−2^) and Capeiti (397 g m^−2^), the tallest of the modern cultivars, than when grown with the other wheat genotypes.

**Table 4:** **Wheat traits measured at plant maturity in 12 genotypes grown in weed-free (WF) and weedy (W) conditions**.

**Genotype**	**Plant height (cm)**		**Lodging (%)**		**Plant biomass (g DM m^−2^)**		**Grain yield (g m^−2^)**		**Harvest Index**		**1000-seed weight (g)**	
	**WF**	**W**	**WF**	**W**	**WF**	**W**	**WF**	**W**	**WF**	**W**	**WF**	**W**
**OLD**												
Biancuccia	129	125	93	83	1192	945	260	162	0.22	0.17	37.2	38.0
Maiorcone	128	127	82	78	1254	1053	240	192	0.20	0.18	38.1	37.3
Realforte	129	125	84	64	1257	1061	262	211	0.21	0.20	38.8	37.6
Russello	155	150	65	41	1299	1054	264	189	0.21	0.18	46.2	44.1
Scorsonera	150	146	43	23	1228	941	287	192	0.24	0.20	46.0	43.6
Cappelli	144	144	88	44	1318	1145	305	245	0.23	0.22	52.4	50.5
**MODERN**												
Capeiti 8	110	106	55	41	1144	725	410	187	0.35	0.26	44.6	39.4
Creso	81	76	0	0	1196	575	424	135	0.35	0.24	51.1	42.1
Simeto	77	75	0	0	1069	511	437	146	0.41	0.29	54.2	45.5
Valbelice	119	115	48	17	1205	789	455	234	0.38	0.30	45.7	40.3
Iride	79	73	0	0	1073	496	452	126	0.42	0.26	42.7	33.6
Claudio	87	85	0	0	1267	605	481	172	0.38	0.28	46.2	41.4
**SIGNIFICANCE LEVEL**												
Genotype (G)	^***^		^***^		^***^		^***^		^***^		^***^	
Interspecific Compet. (IC)	^**^		^***^		^***^		^***^		^***^		^***^	
G × IC	ns		^**^		^***^		^***^		^***^		^***^	
LSD_0.05_	9.4		21.6		132.4		39.4		0.028		2.93	
Old vs. Modern	^***^		^***^		^***^		^***^		^***^		ns	
(Old vs. Modern) × IC	ns		ns		^***^		^***^		^***^		^***^	

*Data are the means of two growing seasons*.

*,

**, *** *significant at the 0.05, 0.01, and 0.001 probability level, respectively*.

*ns, not significant*.

### Grain protein content and yield, N uptake, and recovery of ^15^N fertilizer

Significant differences among wheat genotypes were observed in grain protein content, with the old genotypes having the highest values (on average 13.7 vs. 11.7% for the modern varieties). When a competitor was absent, grain protein yield was significantly higher for the modern varieties than for the old ones (514 and 369 kg ha^−1^ on average, respectively; Table 5); total N uptake ranged from 98 kg N ha^−1^ (Claudio) to 112 kg N ha^−1^ (Cappelli), and recovery of ^15^N fertilizer ranged from 30.0% (Scorsonera) to 36.7% (Biancuccia). Interspecific competition caused a general decrease in grain protein yield, N uptake, and N recovery but with differing intensities among genotypes. In particular, the presence of a competitor resulted on average in a reduction in grain protein yield of 27% in old genotypes and 62% in modern varieties; a reduction in N uptake of 14% in old genotypes and 45% in modern varieties; and a reduction in the recovery of ^15^N fertilizer of 18 and 47% for the old and modern genotypes, respectively. Decreases in both N uptake and recovery of ^15^N fertilizer were associated with proportional increases in both parameters in the competitor; in fact, they were greater when the surrogate weed grew with the modern varieties than when it grew with the old genotypes.

**Table 5:** **Grain protein content and yield, total aboveground N uptake, and ^15^N fertilizer recovery fraction (%^15^N_REC_) in 12 genotypes grown in weed-free (WF) and weedy (W) conditions**.

**Genotype**	**Grain protein content (%)**		**Grain protein yield (kg ha^−1^)**		**Total N uptake (kg N ha^−1^)**			**%15 N_REC_**		
	**Wheat**		**Wheat**		**Wheat**		**Weed**	**Wheat**		**Weed**
	**WF**	**W**	**WF**	**W**	**WF**	**W**		**WF**	**W**	
**OLD**										
Biancuccia	14.6	14.1	379	228	111.0	82.6	19.2	36.7	26.0	7.2
Maiorcone	13.6	14.0	327	268	103.0	91.2	12.0	36.1	27.1	4.7
Realforte	13.1	13.7	343	289	102.0	94.5	12.8	35.6	29.8	4.0
Russello	13.5	13.6	357	257	104.0	94.6	15.6	36.2	28.8	6.2
Scorsonera	14.1	13.6	404	261	100.0	83.6	27.3	30.0	25.9	7.2
Cappelli	13.2	13.0	403	320	112.0	99.0	15.0	34.9	33.3	4.1
**MODERN**										
Capeiti 8	11.8	11.4	485	212	105.5	71.7	33.3	33.6	24.1	10.9
Creso	12.3	13.5	520	182	98.5	46.4	43.6	36.5	15.5	13.8
Simeto	12.0	11.6	523	169	100.0	43.5	51.8	31.4	13.6	17.7
Valbelice	11.7	11.4	532	267	102.0	76.0	30.6	31.6	22.9	8.7
Iride	10.9	11.6	493	146	100.5	42.2	47.8	32.5	12.9	17.2
Claudio	11.1	11.8	533	202	98.0	49.7	45.4	32.0	15.8	13.7
**SIGNIFICANCE LEVEL**										
Genotype (G)	^***^		^*^		^***^		^***^	^***^		^***^
Interspecific Compet. (IC)	ns		^***^		^***^		—	^***^		—
G × IC	ns		^***^		^***^		—	^***^		—
LSD_0.05_	1.41		64.2		17.2		8.53	6.12		3.27
Old vs. Modern	^***^		^*^		^***^		^***^	^***^		^***^
(Old vs. Modern) × IC	ns		^***^		^***^		—	^**^		—

*For total N uptake and %^15^N_REC_ weed data are also reported. Data are the means of two growing seasons*.

*, **, *** *significant at the 0.05, 0.01, and 0.001 probability level, respectively*.

*ns, not significant*.

### Correlations

At tillering time, ground cover was the trait most correlated with variation in yield (both grain and biomass) and weed biomass (*r*-values always greater than |0.80|; Table 6); leaf habit and LAI were positively correlated with variation in wheat grain yield. At heading time, only plant height and plant biomass were significantly (and positively) correlated with variation in biomass and grain yield. Variation in both grain and biomass yield were significantly correlated with N uptake but not with the recovery of ^15^N fertilizer.

**Table 6:** **Simple correlation coefficients among traits measured in wheat genotypes grown in the absence of interspecific competition at tillering, heading, and plant maturity; differences (δ) between weed-free and weedy crops in grain and biomass yield; and weed biomass (*N* = 12)**.

**Wheat traits**	**δ Grain yield**	**δ Biomass yield**	**Weed biomass**
**TILLERING**			
Soil cover	0.83	0.83	−0.82
Plant height	0.26	0.30	−0.20
Leaf habit	0.57	0.54	−0.54
Tiller density	−0.26	−0.29	0.29
Biomass	0.47	0.38	−0.36
LAI	0.61	0.52	−0.56
**HEADING**			
Plant height	0.93	0.96	−0.95
Spike density	−0.04	−0.05	0.01
Biomass	0.77	0.78	−0.81
LAI	0.27	0.15	−0.16
**MATURITY**			
Total N uptake	0.58	0.65	−0.63
% ^15^N_REC_	0.41	0.44	−0.50

*LAI, leaf area index; %^15^N_REC_, ^15^N fertilizer recovery fraction*.

## Discussion

The wheat genotypes under study showed large differences in phenological, morphological, and agronomic traits, at tillering, heading, and maturity. In particular, in the absence of interspecific competition, the old genotypes, compared to the modern ones, covered soil more rapidly, had a more prostrate leaf habit and later heading time, were taller and more susceptible to lodging, and produced more biomass and a lower grain yield. These results were expected, considering the main objectives of the breeding conducted in wheat over the past 70 years. On the whole, when wheat was grown in the absence of a competitor, the old genotypes produced a higher grain protein content than the modern varieties. Such differences most likely depended on the strong variations in grain yield among the genotypes and therefore on a concentration effect. A negative relationship between grain yield and grain N concentration has already been observed by many authors (Calderini et al., 1995; Guarda et al., 2004; Motzo et al., 2004; De Vita et al., 2007).

In the absence of interspecific competition, few differences were found among the wheat genotypes studied in terms of total N uptake. This suggests that the ability of wheat to take up N from the soil was influenced little by breeding. Moreover, our result is in agreement with findings of other authors who observed hardly any correlations between total N uptake and year of cultivar release in wheat (Slafer et al., 1990; Calderini et al., 1995; Foulkes et al., 1998) and who ascribed the general increase in N use efficiency due to breeding activity mainly to the improved ability of the new genotypes compared to the old ones to use the assimilated N to increase grain yield rather than to the increased ability to take up soil N (Brancourt-Hulmel et al., 2003) Modern varieties showed a markedly higher grain protein yield than the old genotypes; as only slight variations have been observed among the studied genotypes for total N uptake, this fact confirms that breeding led to an improvement in N partitioning to the grains. Similar results have been found in barley by Abeledo et al. (2008).

The percent recovery of ^15^N fertilizer of the weed-free wheat was on average 33.9%. The values recorded in this experiment are comparable to those obtained in Tunisia by Sanaa et al. (1992), in Syria by Pilbeam et al. (1997), and in Italy by Giambalvo et al. (2010) and by Ruisi et al. (2014). López-Bellido et al. (2006), in a study performed in Spain on durum wheat, reported values of labeled ^15^N fertilizer recovery ranging from 12.7% when applied at sowing to 41.6% when applied as top dressing. In our study, similar to that observed for total N uptake, few differences were found across the 12 genotypes in the recovery of ^15^N fertilizer.

The detrimental effect of weed competition on wheat growth and yield varied significantly by genotype. The reductions were smaller in the old genotypes and larger in the modern genotypes. According to Lemerle et al. (1996), Gill and Coleman (1999), and Lemerle et al. (2001), observed reductions in grain yield were correlated with weed biomass at maturity.

The presence of a competitor resulted on average in a reduction in both N uptake and the recovery of ^15^N fertilizer that was markedly higher in the modern varieties than in the old ones. Such decreases were associated with proportional increases in both of these parameters in the competitor. Thus, the different abilities of the wheat genotypes studied to compete against weeds seem to depend on their different capacities to suppress weeds (measured by the biomass of the weed), reducing the availability of resources (e.g., N, as resulted in this study from ^15^N fertilizer recovery values) to the competitor, rather than their different abilities to tolerate competition (measured by the ability to maintain yield in the presence of weeds relative to the weed-free condition) and reductions in the contested resources. On the whole, the differences in grain yield observed between the old and modern genotypes, which were very high in weed-free conditions, were canceled out when genotypes were grown in the presence of weed competition. Moreover, while in weed-free conditions the modern varieties showed a grain protein yield markedly higher than the old ones, in weedy conditions the opposite was true. The latter result confirms that the old genotypes were more capable than the modern varieties to compete with weeds for N. These facts could be of interest when one is choosing which variety of durum wheat to grow, and it is particularly important for low-input or organic systems in which weed control is often a serious problem. Moreover, the use of genotypes with high weed suppression ability, reducing weed seed dissemination, can offer a clear advantage to subsequent crops, thus improving the overall efficiency of the cropping system.

Moreover, the results of the present study showed a positive correlation between wheat plant stature at maturity and competitive ability against weeds, as already reported by many authors (Ogg and Seefeldt, 1999; Gonzalez Ponce and Santin, 2001). It is interesting that in the present research, the effects of the interspecific competition on plant growth were already evident at tillering time, when wheat genotypes showed no differences in plant height. Thus, it is clear that other factors must have contributed to determining the different abilities of the wheat genotypes to compete against weeds at tillering time. These were mainly the LAI and the leaf habit, which both influence the percentage of soil surface covered by the crop and, as a consequence, the interception of photosynthetically active radiation captured by weeds. Moreover, Bertholdsson (2005) found in wheat that allelopathic activity can help explain differences among genotypes in competitive ability against weeds in the first phase of the crop cycle. Also, the same author (Bertholdsson, 2004) found higher allelopathic activity higher in old genotypes of barley than in modern ones, thus highlighting an alarming negative evolutionary trend over the past 100 years with respect to this trait. In our research, variations in both grain and biomass yield were significantly correlated with N uptake, thus highlighting the fact that the ability to take up N from the soil could have played a certain role in determining the different competitive abilities against weeds among the genotypes. Hence, it is likely that not one but several factors could have determined the different competitive abilities found among the wheat genotypes and that their importance was variable during the different phases of the crop cycle.

In conclusion, the results of the present study indicate great variations in competitive ability against weeds among the wheat genotypes tested. Overall, the old genotypes showed a higher capacity for suppressing weeds than the modern ones due to a higher ability to utilize resources (e.g., N) when they were contested. This greater ability was positively related to the percentage of soil cover and leaf area at tillering, plant height at heading, and N accumulation capacity, all traits that were expressed more in the old genotypes than the modern ones, inasmuch the most competitive modern genotypes, Capeiti and Valbelice, both had some characteristics in common with the old genotypes. These results could have practical implications for cultivar choice, particularly in organic farming, in which weed control is often a serious problem, and given that the use of a variety high in competitiveness with weeds could have positive effects on subsequent crops due to reductions in the soil weed seed-bank.

### Conflict of interest statement

The authors declare that the research was conducted in the absence of any commercial or financial relationships that could be construed as a potential conflict of interest.
