# Supplementary material for: Nitrogen uptake and nitrogen fertilizer recovery in old and modern wheat genotypes grown in the presence or absence of interspecific competition
Source: Front Plant Sci. 2015 Mar 25;6:185. doi: 10.3389/fpls.2015.00185 (PMC4373253; doi:10.3389/fpls.2015.00185)
Supplement: Supplementary file 1 [file Table1.DOCX]

Table S1. Some morpho-agronomic traits of the wheat genotypes used in the study.

| Genotypes | | Year of release | Group | Lodging susceptibility | 1000-seed weight | Grain yield potential | Grain protein content | Ear color |
| --- | --- | --- | --- | --- | --- | --- | --- | --- |
| 1 | Biancuccia | — | Old | Very high | Low | Very low | Very high | Black/Yellow |
| 2 | Maiorcone | — | Old | High | Very low | Very low | Medium-high | Yellow |
| 3 | Realforte | — | Old | High | Low | Very low | High | Black/Yellow |
| 4 | Russello | — | Old | High | Medium | Low | High | Reddish |
| 5 | Scorsonera | — | Old | High | Medium | Very low | High | Black |
| 6 | Cappelli | 1915 | Old | High | High | Low | High | Black |
| 7 | Capeiti 8 | 1955 | Modern | Medium | Medium | Medium | Medium-high | Black |
| 8 | Creso | 1974 | Modern | Low | Medium-high | Medium-high | Medium-high | Brown |
| 9 | Simeto | 1988 | Modern | Low | High | High | High | Black |
| 10 | Valbelice | 1992 | Modern | Medium | Medium-low | Medium-high | Medium-high | Yellow |
| 11 | Iride | 1996 | Modern | Low | Medium-low | High | Medium-low | Black |
| 12 | Claudio | 1998 | Modern | Low | Medium-high | High | Medium-high | Black |
